# Identification of key gene modules for human osteosarcoma by co-expression analysis

**DOI:** 10.1186/s12957-018-1381-y

**Published:** 2018-05-02

**Authors:** Jing Zhang, Qing Lan, Jiasheng Lin

**Affiliations:** Department of Orthopedics, Fifth Hospital of Harbin, No. 27 Jiankang Road, Xiangfang District, Harbin, 150027 China

**Keywords:** Osteosarcoma, Co-expression, Modules, Gene expression, Function

## Abstract

**Background:**

Osteosarcoma is a type of bone cancer casting huge threat to the human health worldwide. Previously, gene expression analyses were performed to identify biomarkers for cancer; however, systemic co-expression analysis for osteosarcoma is still in need. The aim of this study was to construct a gene co-expression network that predicts clusters of candidate genes associated with the pathogenesis of osteosarcoma.

**Methods:**

Here, we extracted the large scale of datasets from the GEO database. With systematical approaches, we identified the co-expression modules by using weighted gene co-expression network analysis (WGCNA) and investigated the functional enrichments of important modules at GO and KEGG terms.

**Results:**

First, seven co-expression modules, which contain different genes, were conducted for 2228 genes in the 22 human osteosarcoma samples. Then, correlation study showed that the hub genes between pairwise modules displayed great differences. Lastly, functional enrichments of the co-expression modules showed that the module 5 enriched in immune response, antigen processing, and presentation, which is in consistence with GO result. Therefore, we speculated that the module 5 may play a key role in the pathogenesis of osteosarcoma.

**Conclusions:**

Here, we speculated that genes of the module 5 were the essential genes that were associated to human osteosarcoma. Together, our findings not only provided outline of co-expression gene modules for human osteosarcoma, but also promoted the understanding of these modules at functional aspects.

## Background

Osteosarcoma (OS), the most common primary bone malignancy, has an overall incidence of 0.2–3/100000 per year. In the age group of 15–19 years, osteosarcoma is even more common with an incidence of 0.8–11/100,000 per year globally [1, 2]. Despite its rarity, it was also reported as the third most common cancer in adolescence, occurring only less frequently than brain tumor and lymphomas in this age group. Usually, the incidence increases to a peak along with the pubertal growth spurt with gender bias (occurs earlier in females than in males). Besides, tall stature and high birth weight are also reported to be important risk factors [3]. Although the introduction of effective chemotherapy has improved 3-year survival from 20% to 60–70%, no further improvements have been achieved in the last few decades [4]. Therefore, better understanding of genetic etiology and pathology of OS may provide new possible treatment strategies for this tumor.

Several studies have reported that common genetic variations were preliminarily associated with the occurrence of osteosarcoma in some biological pathways, such as TGFBR1*6A, which is a common mutation of TGF-β receptor 1 and was reported to be associated with the distant metastasis of osteosarcoma [5]. Recently, Savage et al. suggested that two loci in the *GRM4* gene at 6p21.3 and in the gene desert at 2p25.2 may be involved in the mechanisms underlying susceptibility to osteosarcoma [6]. However, only a handful of candidate genes are considered to be crucial in the pathogenesis of OS, and there is still a large part needed to be explored.

In some computational research, disease risk modules have been developed to provide significant measurement for cancer diagnosis and to develop novel treatment strategies [5, 7–10]. The weighted gene co-expression network analysis (WGCNA) is a powerful approach based on “guilt-by-association.” It is used to identify gene modules which are popularly applied as candidate biomarkers or therapeutic targets [11, 12]. As a systematical biology method, it was widely used in many complex diseases, such as breast cancer [13], schizophrenia [14, 15], and intracranial aneurysm [16]. By using WGCNA, we are able to construct co-expression networks to detect the differentially correlated gene clusters and perform gene-specific analysis [17, 18].

In this study, WGCNA was constructed based on a dataset comprising 2228 genes from 22 human osteosarcoma samples. The correlation between each module and the biologic functions of genes detected in these modules are analyzed. These informative genes found in our study may be beneficial to clinical treatment of osteosarcoma.

## Methods

### Data processing

Datasets for WGCNA related to osteosarcoma were obtained from the NCBI Gene Expression Omnibus (GEO) (http://www.ncbi.nlm.nih.gov/geo) with accessing number GSE12512. The combined dataset consists of 22 samples. We firstly mapped the array probes to their respective gene IDs by using the array annotations. Probes matching multiple genes were removed from the dataset, and then, we calculated the average expression values of genes measured by multiple probes. A proper threshold was settled based on the amount of genes filtered out.

### Co-expression networks and modules

The influence of power value on the scale independence and mean connectivity were analyzed by using the function *softConnectivity* in WGCNA package. The “randomly selected genes” parameter was set as 5000; other parameters’ set was default. The power parameter was pre-calculated with the function *pickSoftThreshold* in WGCNA. In this function, an appropriate soft-thresholding power for network construction was provided by calculating the scale-free topology fit index of several powers. That is, if the scale-free topology fit index for the reference dataset exceeded 0.8 for low powers (< 30), then the topology of the network is scale-free without batch effects [12]. Next, we summarized the expression values by using the function *collapseRows* in the R package. Cluster analysis was subsequently performed by *flashClust* [11]. The interactions (correlations) of each module was analyzed and visualized by heat map.

### Hub genes and the functional annotations

We performed a gene ontology (GO) enrichment analysis for top 5 modules with most genes by the Database for Annotation, Visualization, and Integrated Discovery (DAVID https://david.ncifcrf.gov/summary.jsp) [19]. Functional enrichment analysis of the hub genes were carried out at GO terms and KEGG pathways (*p* < 0.05) [20, 21]. Before assigning enrichment score for each cluster to make interpretation of the results more straightforward, functional annotation clustering combines single category with a significant overlap in gene content.

## Results

### Pre-processing of the osteosarcoma datasets

**Fig. 1:**
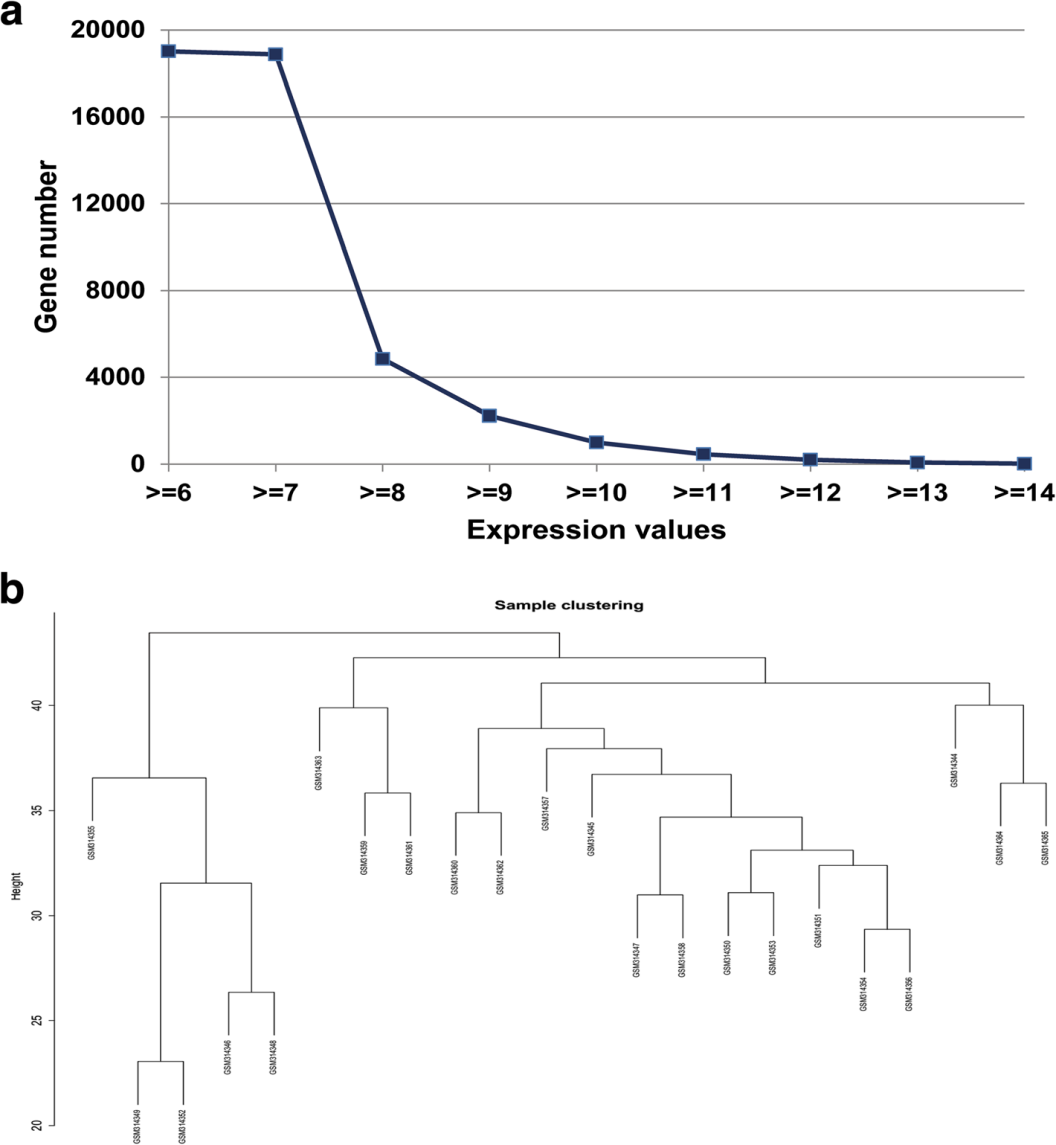
**a** Gene numbers corresponding to different gene expression thresholds. **b** Hierarchical clustering of hub genes in the clustering analysis. Branches of the dendrogram (the meta-modules) represent correlated-positive hub genes

To generate gene co-expression networks, the raw gene expression of osteosarcoma datasets were downloaded from the GEO data repository (http://www.ncbi.nlm.nih.gov/geo). The combined dataset (GSE12512) contained a total of 22 classic OS samples (https://www.ncbi.nlm.nih.gov/geo/query/acc.cgi?acc=GSE12512), and the microarray platform is GPL7192. Then, we identically pre-processed the raw data from every microarray dataset for background correction and normalization. Firstly, probes matching multiple genes were removed out from these datasets, and secondly, the average expression value of gene measured by multiple probes was calculated as the final expression value. Finally, we identify in total 19,015 genes that were expressed. Hereafter, we plotted the relation of gene numbers and gene expression values (Fig. 1) and found that the lowest value is 6.9 and the highest is 14.8. Since the WGCNA was restricted to 3600 genes, we chose the genes of which expression values are larger than 9. In total, 2228 genes were filtered out based on the requirement, which processed 11.7% of the total gene amount.

**Fig. 2:**
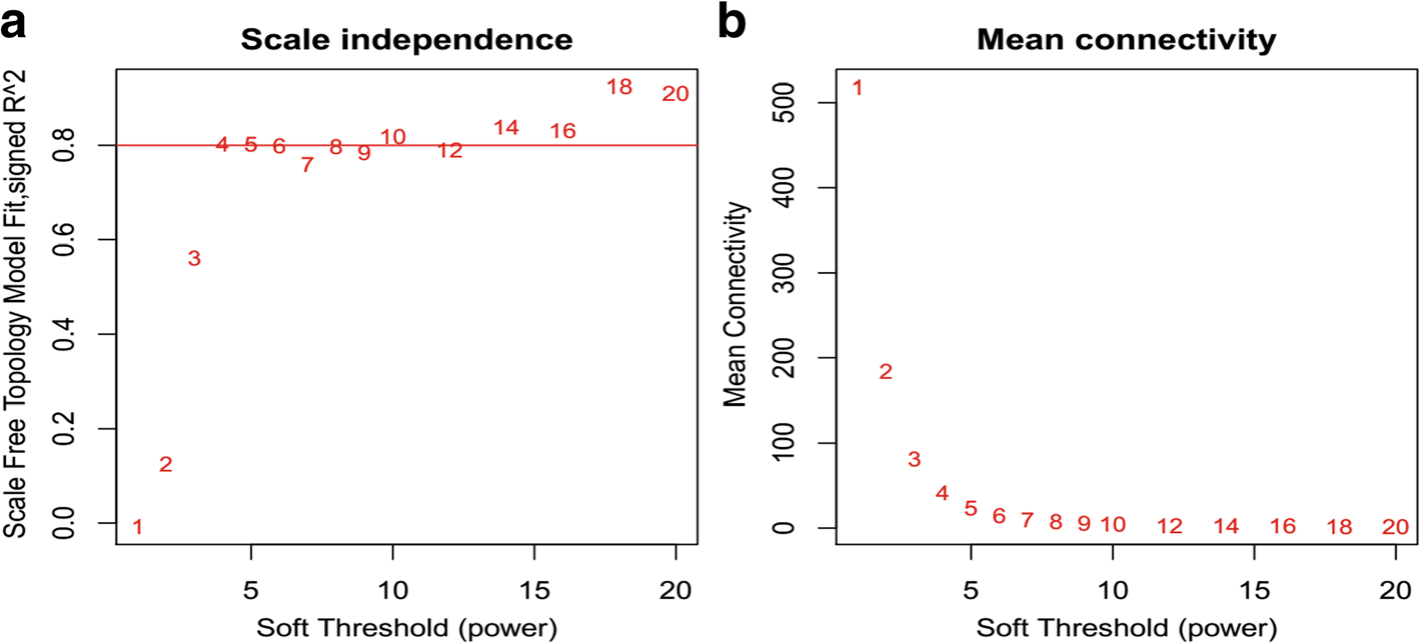
**a**, **b** Network topology of different soft-thresholding powers. The left panel displays the influence of soft-thresholding power (*x*-axis) on scale-free fit index (*y*-axis). The right panel shows the influence of soft-thresholding power (*x*-axis) on the mean connectivity (degree, *y*-axis)

The 2228 genes were further investigated as input for hierarchical clustering analysis, which was performed with the function *flashClust*. We found that these 22 samples mainly yielded two clusters (Fig. 2a), where GSM314346, GSM314348, GSM314349, GSM314352, and GSM314355 became one cluster; the other 17 samples yielded the other one.

### Identification of gene co-expression networks and modules

**Fig. 3:**
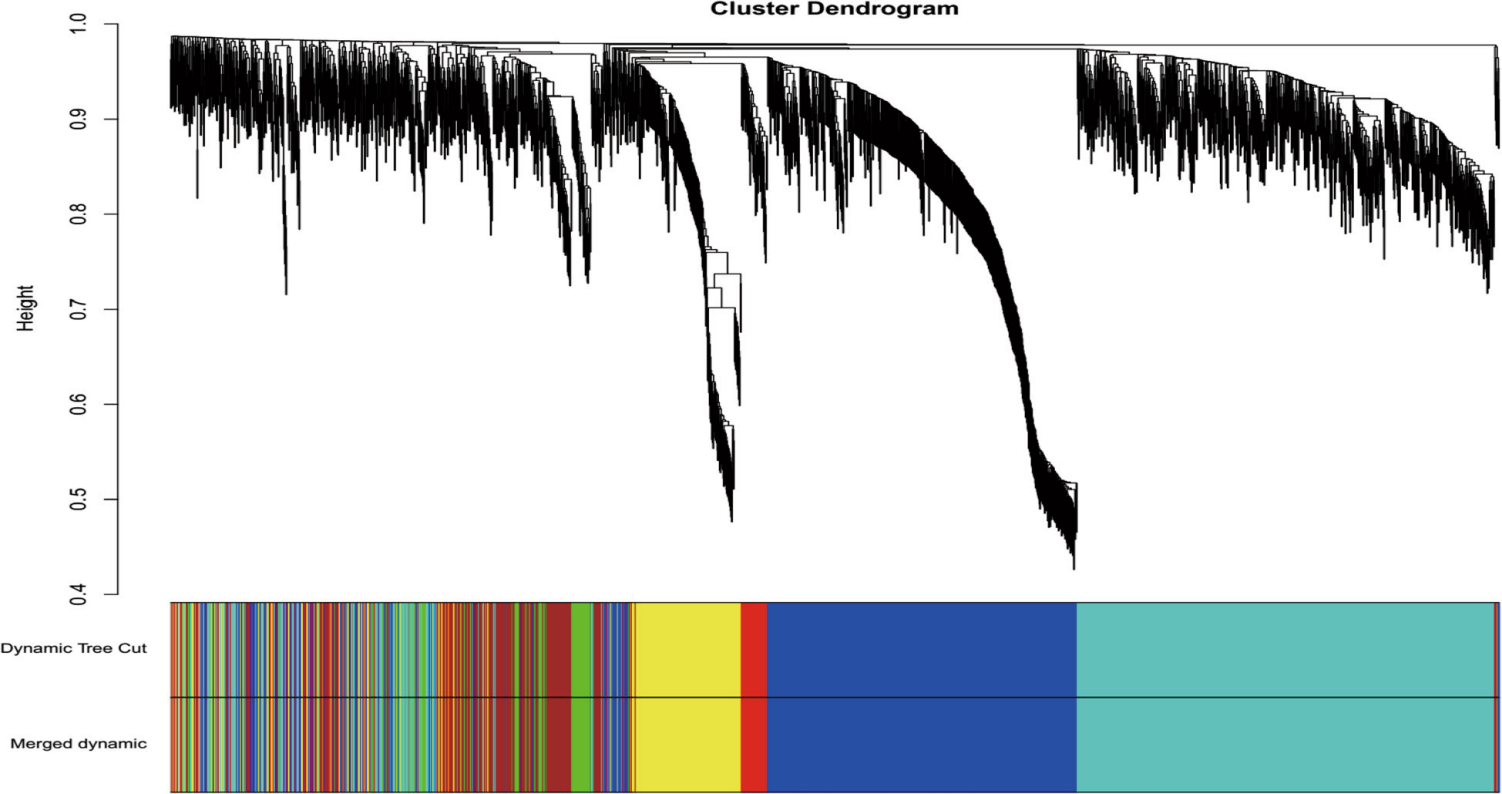
Clustering of genes together with assigned module colors. The dissimilarity was based on topological overlap. The *y*-axis is the distance determined by the extent of topological overlap

The choice of the soft-thresholding power is necessary to construct a WGCNA, to which co-expression similarity is raised to calculate adjacency. Prior to WGCNA conducted to further study the 2228 genes obtained from the 22 samples discussed above, we first performed the analysis of network topology for various soft-thresholding powers in order to have relative balanced scale independence and mean connectivity of the WGCNA. As shown in Fig. 2, power 4, the lowest power for which the scale-free topology fit index reaches 0.90, was chosen to produce a hierarchical clustering tree (dendrogram) of the 2228 genes (Fig. 3). Seven modules were generated and labeled 1–7 from largest to smallest. The largest module contained 838 genes, while the smallest contains 318 genes, and averagely, each module contained 318 genes.

### Correlation between each modules

**Fig. 4:**
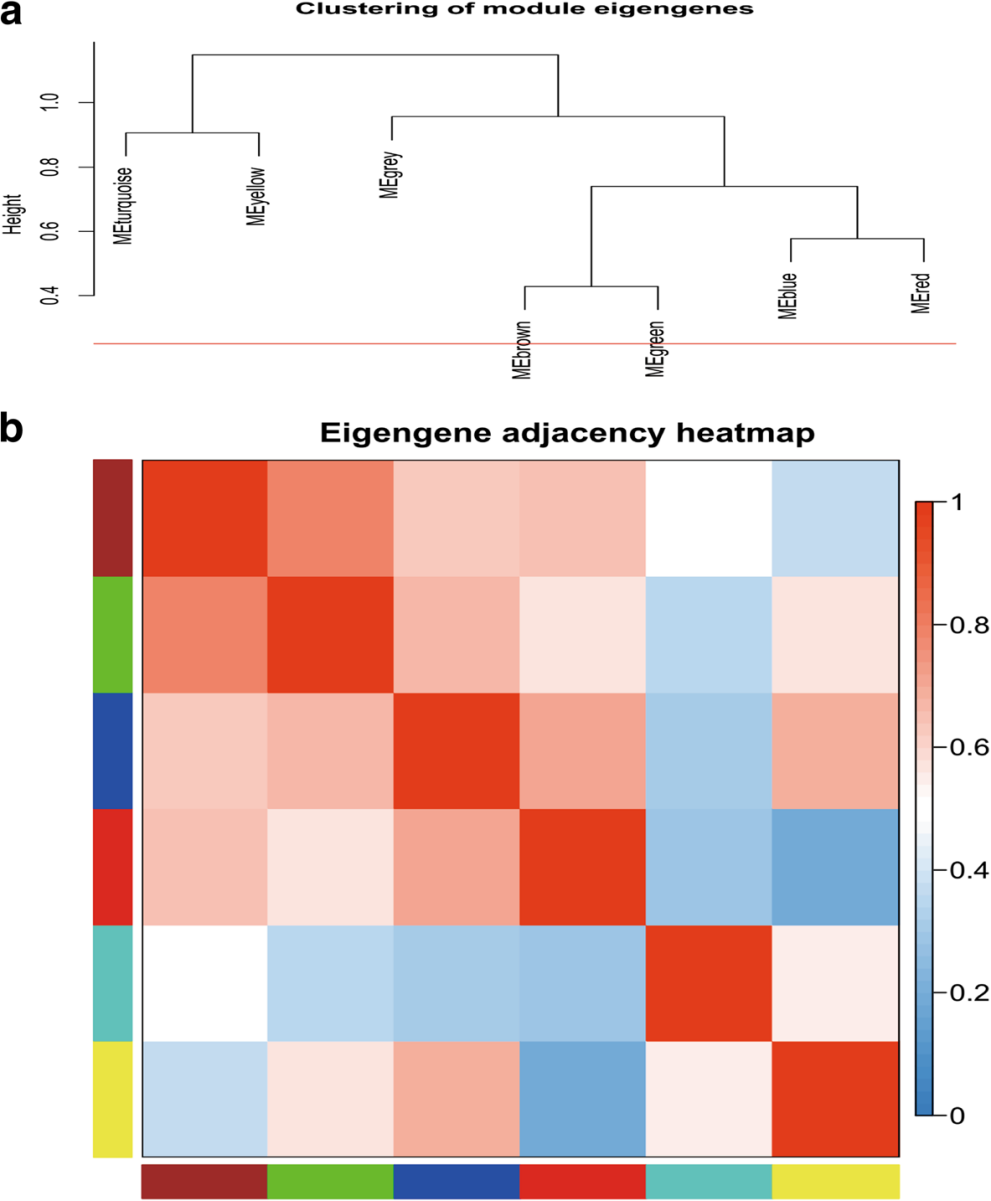
**a** Hierarchical clustering of module hub genes in the clustering analysis. Branches of the dendrogram group together with hub genes that are positively correlated. **b** Heatmap plot of the adjacencies in the hub gene network. The trait weight was included. Each column and row corresponds to one module hub gene (labeled by color) or weight. In the heatmap, red represents high adjacency (positive correlation) and blue represents low adjacency (negative correlation). Red squares along the diagonal are the meta-modules

Based on the network heatmap plot, each module showed independent validation to each other. Therefore, we calculate and cluster the eigengenes of entire modules on their correlations to further quantify co-expression similarity (Fig. 4a). These seven modules yielded two main clusters; one contained two modules, while the other contained the other five modules which can also be divided into three sub-clusters. This result was also supported by the heatmap plot of the adjacencies (Fig. 4b).

### Functional enrichment and clustering analysis

Gene ontology (GO) enrichment analysis for the first five largest network modules was performed by using the Database for Annotation (Table 1), Visualization and Integrated Discovery (DAVID, http://david.abcc.ncifcrf.gov/). Supported by the network heatmap plot, each module had great difference with each other. Genes in module 1 were mainly enriched in cell cycle and negative regulation of cellular protein, especially protein ubiquitination, modules 2 and 4 were enriched in translation process, and module 3 was enriched in energy synthesis while module 5 was enriched in antigen processing and immune response.

**Table 1:** GO enrichment analysis in co-expression modules

	Term	Gene counts	Percent	*P* value	Benjamini
Module 1	Cell cycle	66	0.8	1.30E−10	3.10E−07
	Negative regulation of cellular protein metabolic process	28	0.4	2.80E−10	3.50E−07
	Negative regulation of protein metabolic process	28	0.4	6.80E−10	5.60E−07
	Negative regulation of protein ubiquitination	18	0.2	7.50E−10	4.70E−07
	Anaphase-promoting complex-dependent proteasomal ubiquitin-dependent protein catabolic process	16	0.2	7.10E−09	3.50E−06
Module 2	Translational elongation	51	0.8	4.20E−53	8.70E−50
	Translation	61	0.9	2.60E−34	2.70E−31
	Generation of precursor metabolites and energy	30	0.5	2.00E−09	1.40E−06
	Oxidative phosphorylation	17	0.3	3.40E−09	1.70E−06
	Ribosomal large subunit biogenesis	6	0.1	2.40E−06	1.00E−03
Module 3	ATP synthesis coupled proton transport	6	0.3	9.40E−05	4.50E−02
	Energy-coupled proton transport, down electrochemical gradient	6	0.3	9.40E−05	4.50E−02
Module 4	Translational elongation	14	0.7	1.40E−11	1.50E−08
	Translation	18	0.9	2.00E−08	1.10E−05
Module 5	Antigen processing and presentation	12	1.7	1.30E−13	8.60E−11
	Antigen processing and presentation of peptide or polysaccharide antigen via MHC class II	8	1.1	1.50E−10	4.70E−08
	Immune response	20	2.8	2.20E−10	4.70E−08
	Antigen processing and presentation of peptide antigen	6	0.8	1.80E−07	2.90E−05
	Antigen processing and presentation of exogenous peptide antigen	4	0.6	1.60E−05	2.10E−03

To verify the result of GO enrichment analysis, KEGG pathways were analyzed on the same modules (Table 2). The first four modules were enriched in proteasome (module 1), ribosome (modules 2 and 4), and cell signaling and lysosome (module 4), while the module 5 was enriched in antigen processing, which is in consistence with GO result. Therefore, we speculated that the module 5, antigen process and immune response, may play a key role in the pathogenesis of osteosarcoma.

**Table 2:** KEGG pathways in co-expression modules

	Term	Gene counts	Percent	*P* value	Benjamini
Module 1	Proteasome	14	0.2	3.90E−07	6.10E−05
Module 2	Ribosome	46	0.7	1.80E−41	2.60E−39
	Parkinson’s disease	24	0.4	6.20E−10	4.50E−08
	Oxidative phosphorylation	24	0.4	8.50E−10	4.10E-08
	Huntington’s disease	26	0.4	2.70E−08	9.90E−07
	Alzheimer’s disease	22	0.3	1.40E−06	4.00E−05
Module 3	Epithelial cell signaling in *Helicobacter pylori* infection	9	0.4	1.80E−05	1.80E−03
	Lysosome	11	0.5	2.80E−05	1.30E−03
	*Vibrio cholerae* infection	8	0.4	4.30E−05	1.30E−03
	Oxidative phosphorylation	9	0.4	1.70E−03	4.00E−02
Module 4	Ribosome	14	0.7	2.80E−11	1.90E−09
Module 5	Type I diabetes mellitus	10	1.4	3.30E−11	2.60E−09
	Antigen processing and presentation	12	1.7	4.20E−11	1.60E−09
	Viral myocarditis	11	1.6	2.00E−10	5.10E−09
	Allograft rejection	9	1.3	3.30E−10	6.50E−09
	Graft-versus-host disease	9	1.3	6.70E−10	1.00E−08

## Discussion

The main objective for this study was to utilize a global approach to construct a gene co-expression network that predicts clusters of candidate genes involved in the pathogenesis of osteosarcoma. We hypothesized that tightly co-expressed gene modules with common functional annotation would be able to predict candidate gene sets that underlies a given biological process.

WGCNA is a relatively novel statistical approach based on gene correlations and has been used not only to construct gene networks and detect modules/sub-networks, but also to identify hub genes and select candidate genes as biomarkers [11]. Usually, module detection in WGCNA needs a knowledge-independent process. However, selection of a threshold for culling the network to limit noise would probably rely on empirical judgment and functional annotation [11]. Furthermore, WGCNA can only provide a set of hub genes instead of specific genes related to the background, such as osteosarcoma in this study. Therefore, further studies should be carried out to narrow down the gene targets. Such as RMT method, this lies in its ability to automatically localize the noise-to-signal threshold instead of using empirical judgment or annotations [22]. Moreover, construction of mutant will also help to understand the role of one or more specific genes in the pathogenesis of osteosarcoma.

**Fig. 5:**
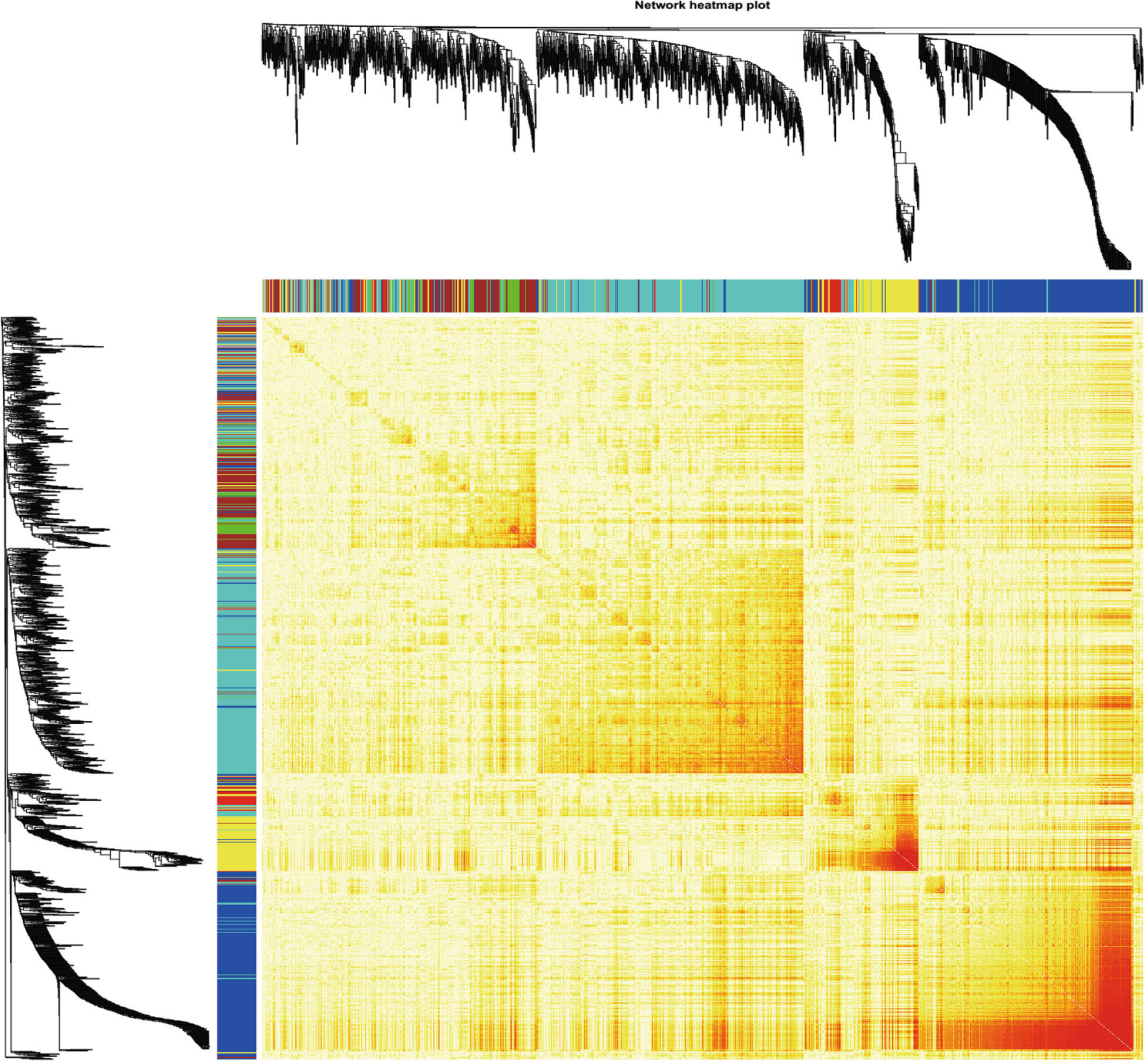
**a**, **b** Network heatmap plot. Branch in the hierarchical clustering dendrograms correspond to each module. Color-coded module membership is showed in the color bars beneath and towards the right of the dendrograms. In this heatmap, the progressively more saturated yellow and red indicate the high co-expression interconnectedness. Modules correspond to highly interconnected gene blocks. Genes of high intra-modular connectivity are located at the top of the module branches. These genes show the highest interconnectedness with the rest of the genes in the module

Here, WGCNA was applied to investigate 2228 genes of 22 samples that were compromised from a dataset obtained from NCBI, and seven modules were yielded. According to correlation study by network heatmap plot (Fig. 5), all the modules have almost no correlation with each other. GO enrichment and KEGG pathway analysis were performed to further study the biological functions of genes enriched in five largest modules. Both GO and KEGG showed that, in consistence with correlation study, no module is involved in the same functions/pathways with each other (Tables 1 and 2). Modules 1–4 were involved in protein ubiquitination, translation process, energy synthesis, etc. But interestingly, the genes in module 5 were consistently involved in antigen processing and immune system in both GO and KEGG result.

Endo Munoz et al. have reported that OS are characterized by an early deregulation of genes involved in antigen presentation and suggest that patient prognosis is determined early in tumor development and that enhancing antigen presentation may be clinically valuable in treating OS [23]. Furthermore, several immune molecules, such as cytotoxic T cell lymphocyte antigen 4 (CTLA4) and CD40 (TNF receptor superfamily 5), have been targeted clinically in osteosarcoma. It was discovered that they can break the immune tolerance in tumor [24]. Therefore, we suggested the genes in module 5 might play a key role in the pathogenesis of osteosarcoma and thereby provide potential targets for treating OS.

## Conclusion

In summary, this research creatively applied transcriptional network analysis to identify co-expression module. In module 5, the highly enriched genes were involved in the antigen and immune process. According to their collective expression, they were speculated to be correlated with pathogenesis of osteosarcoma as well.

The discoveries in this study might be used to predict clusters of candidate genes associated with the pathogenesis of osteosarcoma. This might contribute to improve or optimize clinical diagnosis by using molecular techniques. However, the clinical specific efficiency of the identified module needs more experiments to clarify.
